# Effect of the Gall Wasp *Leptocybe invasa* on Hydraulic Architecture in *Eucalyptus camaldulensis* Plants

**DOI:** 10.3389/fpls.2016.00130

**Published:** 2016-02-15

**Authors:** You-Gui Tong, Xiao-Xi Ding, Kai-Cun Zhang, Xin Yang, Wei Huang

**Affiliations:** ^1^Forestry Bureau of Dongchuan CountyKunming, China; ^2^Kunming Forest Resources AdministrationKunming, China; ^3^Kunming Institute of Botany, Chinese Academy of SciencesKunming, China

**Keywords:** *Eucalyptus camaldulensis*, gall wasp, hydraulic function, *Leptocybe invasa*, photosynthesis

## Abstract

The gall wasp, *Leptocybe invasa* (Hymenoptera; Eulophidae), is a devastating pest of eucalypt plantations in the Middle East, the Mediterranean basin, Africa, India, South-East Asia, and China. Heavy galling causes the leaves to warp and in extreme cases it may stunt the growth of the trees of *Eucalyptus camaldulensis*. However, the physiological mechanisms underlying how *L. invasa* inhibits the growth of plants of *E. camaldulensis* are unclear. Because the growth rate of plants is mainly dependent on photosynthesis that is largely correlated with hydraulic architecture, we speculate that galling of *L. invasa* depresses hydraulic conductance of stem and leaf. In the present study, we examined the effects of *L. invasa* galling on hydraulic architecture and photosynthetic parameters in *E. camaldulensis* plants. We found that galling of *L. invasa* significantly decreased stem hydraulic conductance (*K*_stem_), midday leaf water potential (Ψ_md_), minor vein density, and stomatal density (SD). Furthermore, the stomatal conductance (*g*_s_), chlorophyll content, CO_2_ assimilation rate (*A*_n_) and photosynthetic electron flow were reduced in infected plants. Therefore, the galling of *L. invasa* not only declined the water supply from stem to leaves, but also restricted water transport within leaf. As a result, galled plants of *E. camaldulensis* reduced leaf number, leaf area, SD and *g*_s_ to balance water supply and transpirational demand. Furthermore, galled plants had lower leaf nitrogen content, leading to decreases in chlorophyll content, CO_2_ assimilation rate and photosynthetic electron flow. These results indicate that the change in hydraulic architecture is responsible for the inhibition of growth rate in galled plants.

## Introduction

In China, *Eucalyptus* sp. has been introduced for plantation in many tropical and subtropical areas for economic and social development because they can tolerate and grow well on degraded, unfertile soils, where it is very difficult to establish other tree species. The wood of *Eucalyptus* species can be used for production of paper, which providing income for local people and government. The genus *Eucalyptus* in its native range sustains a rich fauna of gall-inducing insects (Blanche and Westoby, 1995; Blanche, 2000). *Eucalyptus* is also a unique genus in hosting several Eulophidae wasps (Hymenoptera: Chalcidoidea) such as *Leptocybe invasa* Fisher & LaSalle. *Eucalyptus camaldulensis* Dehnh. is one of the most susceptible species to this gall wasp *L. invasa* (Thu et al., 2009). Recently, the massive presence of galls induced by *L. invasa* cause severe damage and consequent economic impact in nurseries and eucalyptus plantations with *E. camaldulensis* (Thu et al., 2009).

*Leptocybe invasa*, a gall-inducing wasp on several *Eucalyptus* sp., was first reported in 2000 from the Middle East and was subsequently found in many countries in the Mediterranean basin, Africa, and Asia (Mendel et al., 2004). The adult of *L. invasa* is very small (1.0–1.4 mm long), and it lays the eggs in the cortex of shoots or the midribs of leaves. The eggs hatch and the larvae feed and then pupate. The adults emerge from the gall within a period of 4–5 months (Mendel et al., 2004). The gall size is correlated to the number of wasps developing in the gall. The developing larvae form typical galls in the form of distinct swellings on the leaf midribs, petioles, and stems on new foliage on trees of all ages (Kim et al., 2008). Seedlings in the nursery and young plantations are particularly susceptible (Thu et al., 2009). Heavy galling causes the leaves to warp and in extreme cases it may stunt the growth of the tree (Mendel et al., 2004). However, the physiological mechanisms underlying why *L. invasa* inhibits the growth of *E. camaldulensis* in unclear.

Field observations show that the presence of *L. invasa* largely decreases the leaf number and leaf area. It has been indicated that leaf water status is one of the most important factor determining the leaf area (Anyia and Herzog, 2004; Koch et al., 2004). Under drought stress, plants decrease leaf area and leaf number to diminish the water transpiration (Anyia and Herzog, 2004; Koch et al., 2004). Therefore, we speculate that the infection of *L. invasa* decreases the leaf water potential. In addition, the growth of plants is dependent on leaf primary productivity (photosynthesis), which in turn relies on the function of hydraulic architecture such as stomatal density (SD) and minor vein density (MVD; Brodribb et al., 2007; Tanaka et al., 2013; Zhu et al., 2013; Hu et al., 2014). Therefore, the severe damage to *E. camaldulensis* plants caused by galling of *L. invasa* is probably lined to change in the function of leaf hydraulic architecture.

Water transport from stem to leaf is a prerequisite for the maintenance of optimum leaf water potential (Chone et al., 2001; Sperry et al., 2002; Zhang and Cao, 2009). The stem hydraulic conductance (*K*_stem_) is determined by the number and radius of functional conduit (Hellemans et al., 1980). Under drought stress, the number of functional conduit decreases due to cavitation, causing the decline in *K*_stem_ (Sperry et al., 2002). Once *K*_stem_ decreases, plants regulate the development of leaf hydraulic architecture to decrease transpirational demand. As mentioned above, the leaf development in the galled plants of *E. camaldulensis* is similar to the phenomenon under drought stress. Therefore, we speculate that the presence of galls in the cortex of shoots maybe leaf to cavitation of stem conduit, which decreases the number of functional conduit and thus *K*_stem_.

To explain, why *E. camaldulensis* plants galled by *L. invasa* display lower growth rate, we determined the difference in hydraulic architecture between galled and non-galled plants. To our knowledge, the effect of *L. invasa* infection on the function of hydraulic architecture in *E. camaldulensis* has never been reported before. The present study aims to test the hypothesis that galling of *L. invasa* decreases the water supply to leaf and then affect the development of leaf hydraulic architecture. Furthermore, the effects of galling of *L. invasa* on stomatal conductance and photosynthetic rate have been studied.

## Materials and Methods

### Plant Materials and Study Site

This study was conducted at Dongchuan County, Yunnan Province, China. *E. camaldulensis* Dehnh. is one of the most susceptible species to the gall wasp *Leptocybe invasa* Fisher & LaSalle. In the present study, galled and non-galled plants of *E. camaldulensis* grown in the same open-field place with sufficient water were used for measurements of *K*_stem_, leaf anatomy, and gas exchange. All measurements were conducted in August 2014 (summer). Three-years-old plants of galled and non-galled plants were used for hydraulic and photosynthetic measurements. The galled-plants were naturally got infected by *L. invasa.* Six independent stems from galled or non-galled plants were used for measurements of *K*_stem_. Nine independent leaves of galled or non-galled plants were used for measurements of leaf anatomy. Four independent leaves of galled or non-galled plants were used for photosynthetic measurements. Immediately after the measurements of gas exchange, leaf samples were taken for measurements of leaf anatomy. Some leaves taken from other plants were also used for measurements of leaf anatomy.

### Hydraulics

Stem hydraulic conductance was determined using a high-pressure flow meter (HPFM; Dynamax Inc., Houston, TX, USA). The high-pressure flow meter measured resistance (the inverse of conductance) as the force required to push water through a sample for a given flow rate. Samples were cut from the trees and wrapped with wet tissue in plastic black bags for transport to the laboratory. Briefly, the stem was recut under water and then connected to the flow meter. Water flowed through the stem and the necessary applied pressure were recorded every 3 s while pressure was ramped. Here, *K*_stem_ was computed as the water flow rate per unit cross-sectional area of the stem divided by the pressure drop. During this measurement period, the laboratory temperature was about 25°C. Leaf water potential was measured on six fully expanded and mature leaves per treatment (galled and non-galled plants). After measuring photosynthesis, leaves were taken and wrapped with wet tissue in plastic black bags for transport to the laboratory. In our present study, leaf water potential was measured with WP4 Dewpoint Potentiometer (Decagon Devices, Inc., Pullman, WA, USA).

### Leaf Anatomy

Leaves were boiled in 7% NaOH for 3 min and then stained with safranin. The sections were then photographed under a light microscope at 4× magnification (Nikon Optiphot; Nikon, Tokyo, Japan). Values for MVD were expressed as the sum of the lengths of third- and higher-order veins per unit area (Zhu et al., 2012). SD was measured on both adaxial and abaxial cuticles. SD was measured from digital photomicrographs of the cuticle preparation at 20× magnification. The data of MVD and SD include six section per leaf, for a total of six leaves per treatment (galled and non-galled plants).

### Gas Exchange Measurement

Parameters for gas exchange and chlorophyll fluorescence were monitored with an open gas exchange system that incorporated infrared CO_2_ and water vapor analyzers (Li-6400XT; Li-Cor Biosciences, Lincoln, NE, USA) and a 2-cm^2^ measuring head (6400-40 Leaf Chamber Fluorometer; Li-Cor Biosciences). The atmospheric CO_2_ concentration was maintained at 390 μmol mol^–1^ by the Li-6400XT with a relative humidity of approximately 50%. To generate a light response curve, we initially exposed the mature leaves to strong irradiance (1500 μmol photons m^–2^ s^–1^) for 15 min to obtain steady, high levels of *g*_s_ and CO_2_ assimilation. Afterward, photosynthetic parameters were evaluated at 2-min intervals at photosynthetic photon flux densities (PPFDs) of 2000, 1600, 1200, 1000, 800, 500, 300, 150, 100, 50, 20, and 0 μmol photons m^–2^ s^–1^. Both photosynthetic rate and stomatal conductance were recorded automatically by Licor-6400XT. The fluorescence parameters *F*_m_′, and *F*_s_ were evaluated as previously described in Baker and Rosenqvist (2004). *F*_m_′ represents the minimum and maximum fluorescence after light-adaption. *F*_s_ is the light-adapted steady-state fluorescence. Effective quantum yield of PSII was calculated as Φ_PSII_ = (*F*_m_′ –* F*_s_)/*F*_m_′ (Genty et al., 1989). Total photosynthetic electron flow through PSII was calculated as ETRII = Φ_PSII_ × PPFD × 0.85 × 0.5 (Krall and Edwards, 1992).

### Chlorophyll Content

The chlorophyll content was measured according to the method of Lichtenthaler and Wellburn (1983).

### Statistical Analysis

The results were displayed as mean values of independent measurements (*n* = 4–9). We used one-way ANOVA and SPSS 16.0 software (SPSS Inc., Chicago, IL, USA) to examine differences between the galled and non-galled plants. Those differences were considered significant at *P* < 0.05.

## Results

**FIGURE 1:**
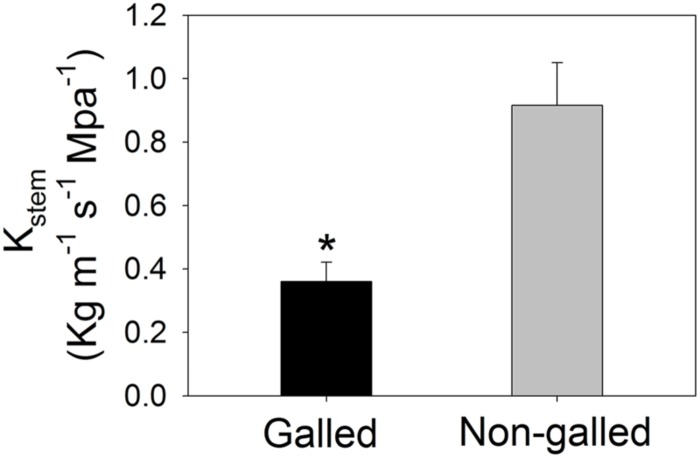
**Stem hydraulic conductance (*K*_stem_) in galled and non-galled plants of *Eucalyptus camaldulensis*.** An asterisk indicates a significant difference between galled and non-galled plants.

**FIGURE 2:**
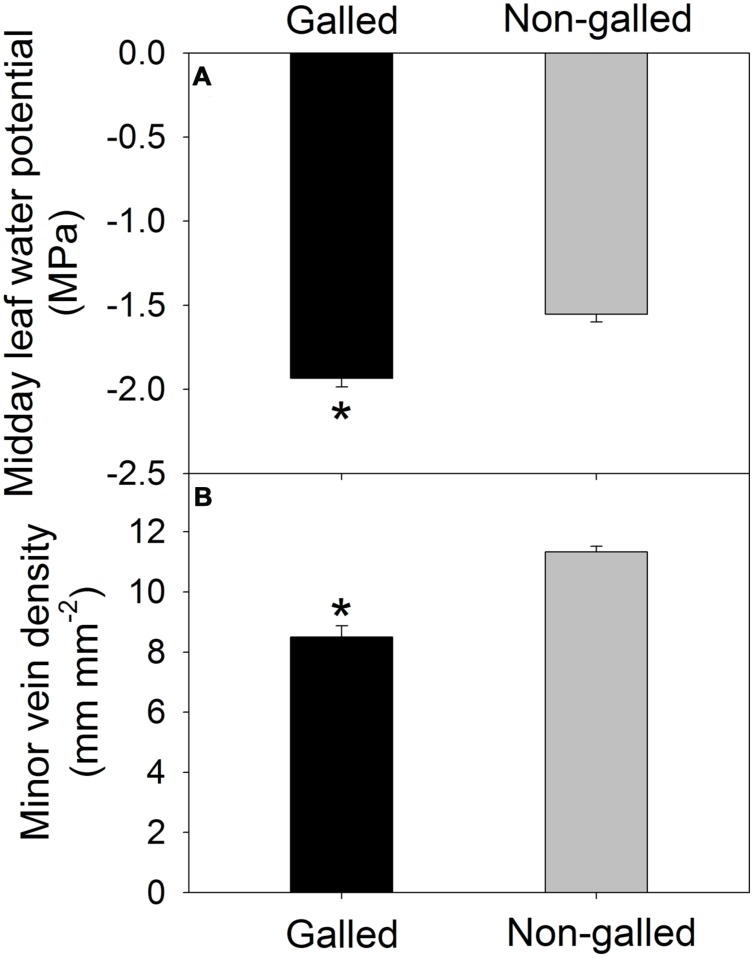
**Midday leaf water potential (A) and minor vein density (B) in galled and non-galled plants of *Eucalyptus camaldulensis*.** An asterisk indicates a significant difference between galled and non-galled plants.

Values for the stem hydraulic conductance (*K_stem_*) were 0.36 and 0.92 Kg m^–1^ s^–1^ MPa^–1^ in galled and non-galled plants, respectively (**Figure 1**). The value of *K_stem_* in galled plants was 60% lower than that in non-galled plants. Therefore, galling in the stems largely decreased *K_stem_*, probably as a result of jam and cavitation of conduit. Meanwhile, the midday leaf water potential (Ψ_md_) was affected by galling of *L. invasa*. The value of Ψ_md_ was significantly lower in galled plants (–1.93 MPa) than non-galled plants (–1.55 MPa; **Figure 2A**). In leaves of galled plants, the MVD largely decreased compared with non-galled plants. The value of MVD was 8.5 mm mm^–2^ in galled plants versus 11.3 mm mm^–2^ in non-galled plants (**Figure 2B**). Because MVD is significantly and positively correlated with leaf hydraulic conductance (*K*_leaf_), the decrease in MVD in galled plants can cause a decline in *K*_leaf_. The lower Ψ_md_ in leaves of galled plants is probably a result of decreases in both *K*_leaf_ and *K_stem_*.

**FIGURE 3:**
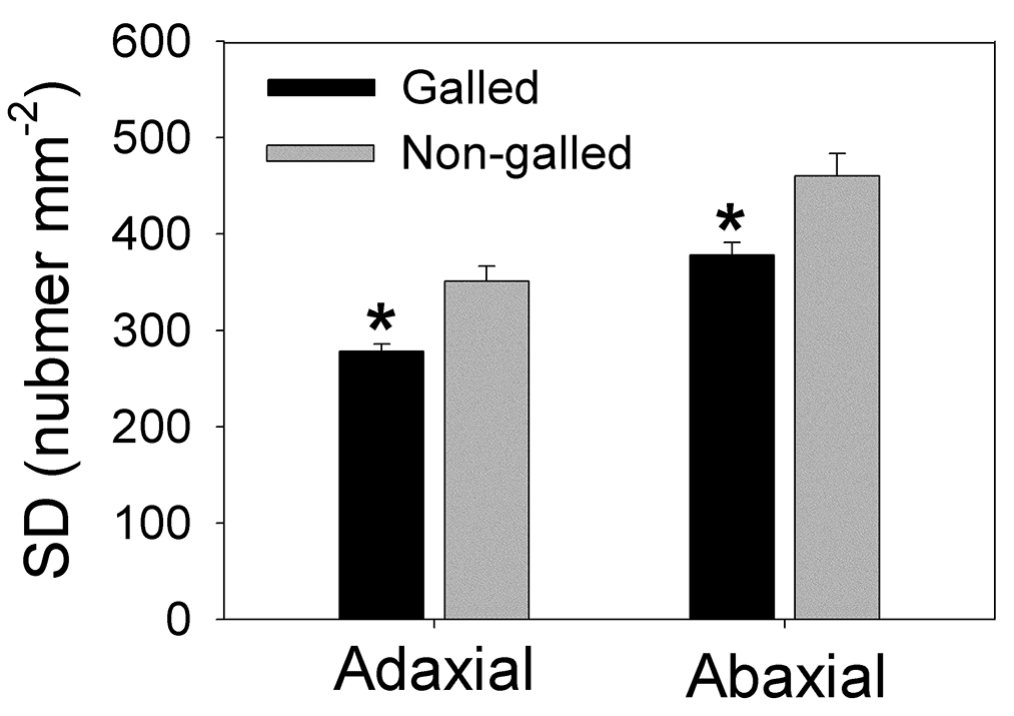
**Stomatal density (SD) in adaxial and abaxial sides in galled and non-galled plants of *Eucalyptus camaldulensis*.** An asterisk indicates a significant difference between galled and non-galled plants.

Stomatal density is another important functional trait that reflects leaf water status and determines stomatal conductance (*g*_s_) and photosynthetic rate (*A*_n_). Stomata exist in both adaxial and abaxial sides in leaves of *E. camaldulensis*, contributing to its high level of photosynthetic rate. Interestingly, galling of *L. invasa* significantly affected the development of stomata. In galled and non-galled plants, SD in adaxial side numbered 278.3 and 351.3 per square millimeter, respectively, and SD in abaxial side numbered 378.2 and 460.3 per square millimeter, respectively, (**Figure 3**). The values of SD in adaxial and abaxial sides in galled plants were approximately 80% of non-galled plants (**Figure 3** and **Supplementary Figure S1**).

**FIGURE 4:**
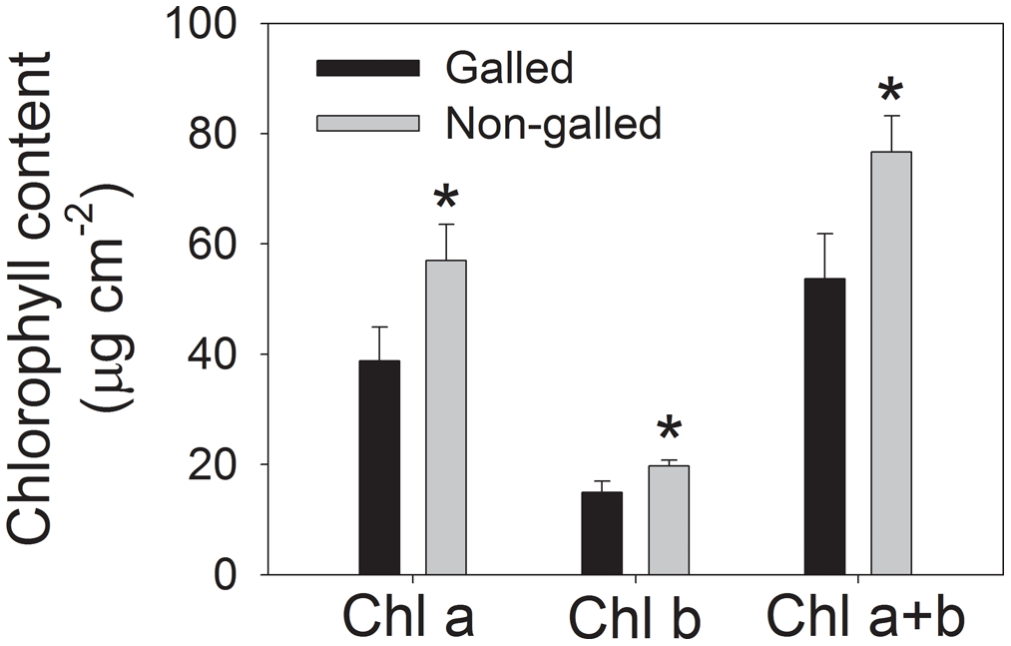
**Chlorophyll content in galled and non-galled plants of *Eucalyptus camaldulensis*.** An asterisk indicates a significant difference between galled and non-galled plants.

**FIGURE 5:**
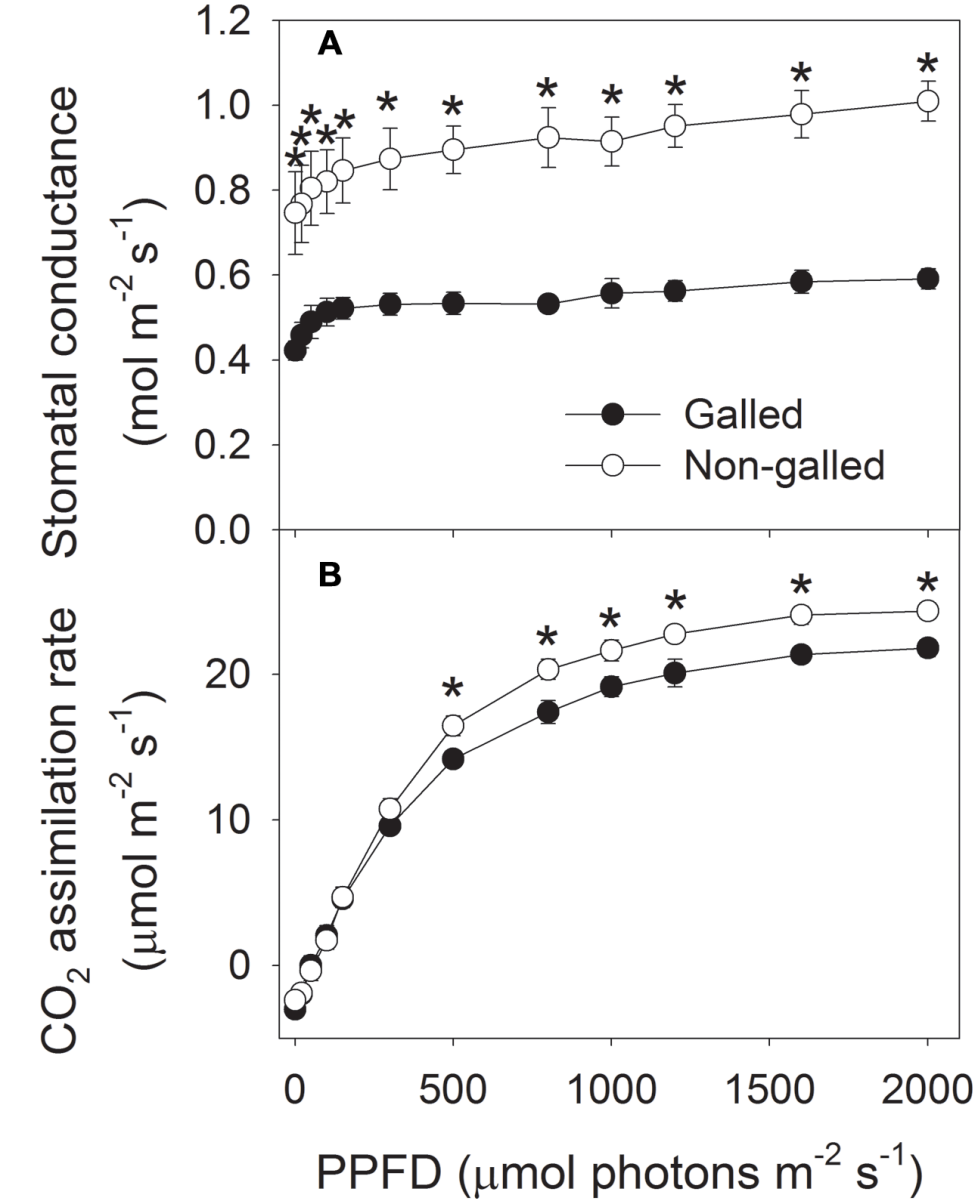
**Responses of stomatal conductance (A) and CO_2_ assimilation rate (B) to incident light intensity in galled and non-galled plants of *Eucalyptus camaldulensis*.** An asterisk indicates a significant difference between galled and non-galled plants.

**FIGURE 6:**
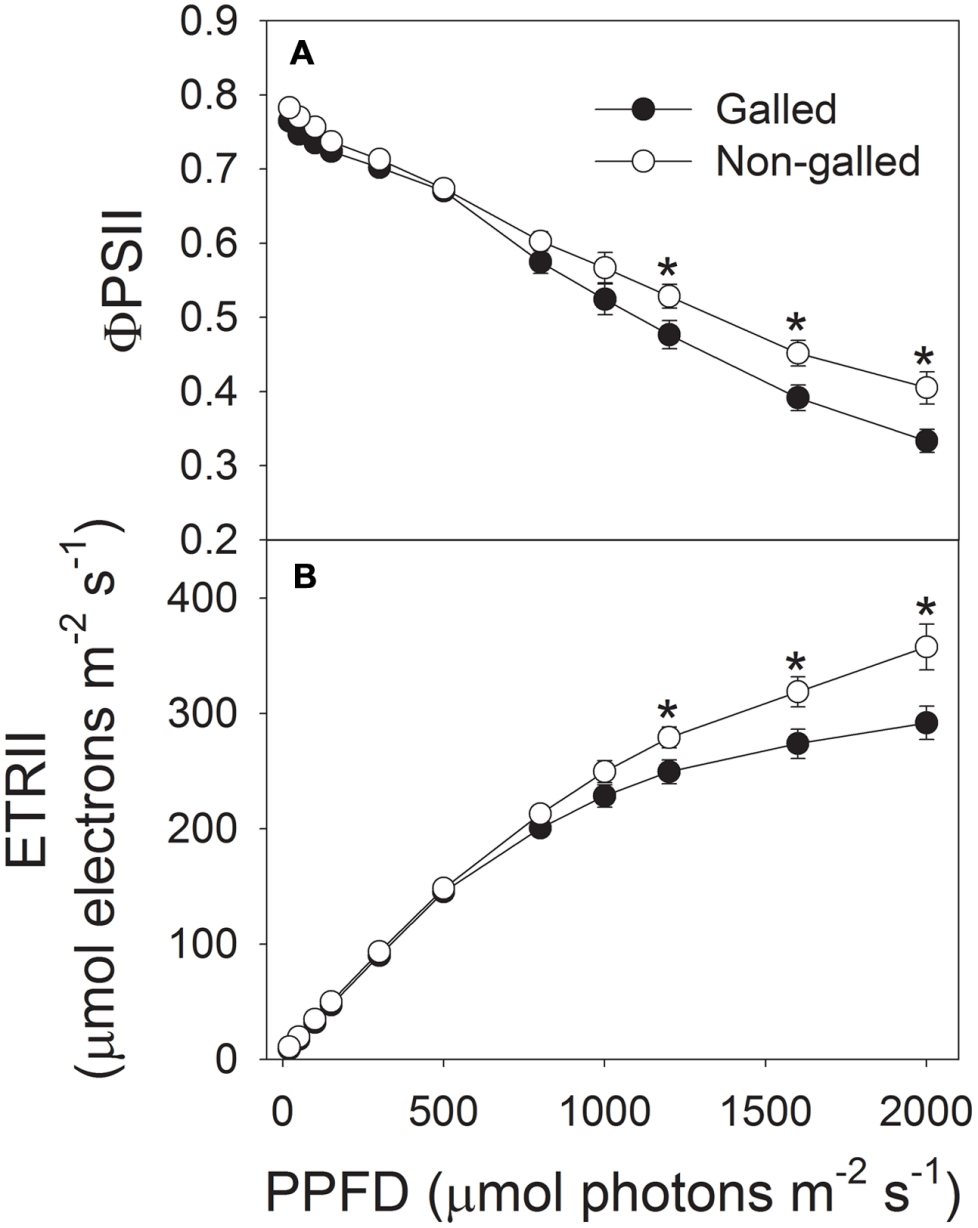
**Responses of effective quantum yield of PSII (Φ_PSII_) (A) and electron flow through PSII (ETRII) (B) to incident light intensity in leaves of galled and non-galled plants of *Eucalyptus camaldulensis*.** An asterisk indicates a significant difference between galled and non-galled plants.

To determine the effect of galling of *L. invasa* on photosynthetic characteristics in *E. camaldulensis*, we measured chlorophyll content and gas change in galled and non-galled plants. The galled plants showed significantly lower chlorophyll content than the non-galled plants (**Figure 4**). Because chlorophyll content is mainly determined by leaf N content, this result suggested that galling of *L. invasa* decreased leaf N content. Light response curves indicated that galling of *L. invasa* significantly affected the values of *g*_s_ (**Figure 5A**). Furthermore, as indicated by light response curves, the maximum rate of CO_2_ assimilation was depressed by the galling of *L. invasa* (**Figure 5B**). As a result, the primary productivity was affected by galling of *L. invasa*. Under light intensities above 1200 μmol photons m^–2^ s^–1^, values for effective quantum yield of PSII (Φ_PSII_) and electron flow through PSII (ETRII) were significantly lower in the galled-plants than non-galled plants (**Figure 6**), further indicating the depression of photosynthesis by the galling of *L. invasa*.

## Discussion

Gall density decreased with maturity of the host plant and was three times higher on juvenile plants compared with mature plants (Pires and Price, 2000). The distribution of attack in relation to plant age was not related to changes in shoot size with the age of the plants (Pires and Price, 2000). An infestation of *L. invasa* can cause stun of growth of the young trees of *E. camaldulensis*. Moreover, severe infestation may result in die-back and death of galled trees (Nyeko et al., 2009; Thu et al., 2009). However, the underlying physiological mechanisms are unclear. Here, we focused on the question whether galling of *L. invasa* affect the change in hydraulic function in *E. camaldulensis*. Particularly, we examined whether gall induction restricts stem hydraulic conductance and subsequently affects leaf functional traits.

We found that galling of *L. invasa* largely decreased the stem water transport in plants of *E. camaldulensis*. Theoretically, *K_stem_* is mainly determined by conduit radius according to Hagen–Poiseuille equation (Hellemans et al., 1980). Conductance through an ideal pipe is proportional to the fourth power of its radius. As a result, a small increase in conduit radius leads to a large increase in vascular hydraulic conductance. The developing larvae of *L. invasa* form typical galls in stems, which hardly changes the conduit radius but can cause the jam of conduit. When the jam of conduit occurred, water transport through conduit would be restricted, leading to the decrease in *K_stem_*. Furthermore, the galls caused by *L. invasa* increase the risk of cavitations of conduit that decline stem hydraulic conductance. Whatever which mechanism, the developing larvae of *L. invasa* largely decreased *K_stem_* in *E. camaldulensis*. It is documented that the gall formation by *L. invasa* on shoots and leaves of *E. camaldulensis* results in quicker abscission of leaves and drying up of shoots and even death of trees (Nyeko et al., 2009; Thu et al., 2009). The data of *K_stem_* presented here provided new evidence that infection of *L. invasa* caused hydraulic limitation in the galled plants of *E. camaldulensis*.

It has been indicated that *K_stem_* plays an important role in determining leaf water status and development of leaf hydraulics (Sack and Frole, 2006; Zhu et al., 2013). Our results indicated that the infected plants of *E. camaldulensis* showed lower midday leaf potential. What is more, the MVD also decreased in the infected plants of *E. camaldulensis*. Because MVD is an important determining factor for leaf hydraulic conductance (*K*_leaf_; Hu et al., 2014), the decrease in MVD can cause a decline *K*_leaf_, which further restricts the water supply to leaf. Therefore, decreases in both *K_stem_* and *K*_leaf_ induced the significant decline in midday leaf water potential in plants galled by *L. invasa*. Meanwhile, comparing with uninfected plants, SD decreased significantly in the infected plants. The parameter SD is corresponding to the potential transpirational demands in leaves. The coordination between MVD and SD allowed the leaves to maintain an efficient balance between water supply and transpiration. The MVD and SD usually develop coordinately in plants. For example, tobacco leaves grown at high temperature had higher values of MVD and SD than plants grown at lower temperature (Hu et al., 2014). Genotypes of *Arabidopsis thaliana* in which mature leaves have lower MVD tend to have reduced SD (Pérez-Pérez et al., 2011). In *Paphiopedilum* (Orchidaceae), evolutionary association of stomatal traits with leaf vein density is found (Zhang et al., 2012). Taking together, the declined SD in the galled plants diminished transpirational rate, thereby balancing water supply with transpirational demand.

In the infected plants of *E. camaldulensis*, mature leaves showed much lower *g*_s_, as indicated by light response curves. Stomatal conductance is a complex process that can be affected by several factors, such as leaf ABA content (Tardieu et al., 1991, 1992, 1996), SD (Tanaka et al., 2013), leaf water potential (Tardieu et al., 1996; Comstock and Mencuccini, 1998; Zhu et al., 2009; Choat et al., 2012), stem hydraulic conductance (Brodribb et al., 2007; Zhu et al., 2013), and leaf hydraulic conductance (Brodribb et al., 2007; Zhu et al., 2013). Because ABA content changed slightly between galled and non-galled plants (data not shown), the lower *g*_s_ in the infected plants was independent on ABA content. In the galled plants, the decline in *K_stem_* and *K*_leaf_ restricted water supply to leaves and caused a decrease in leaf water potential. Under such conditions, leaves of the galled plants closed stomata to reserve water and avoid severe water deficit. Furthermore, the decrease in *g*_s_ can avoid over-cavitation of stem conduit (Zhang et al., 2013). Therefore, for the galled plants, the lower *g*_s_ is an adaptive strategy to control transpiration in response to the changes in stem and leaf hydraulic conductance.

Gall formation has been found to significantly modify foliar gas exchange processes (Fay et al., 1993; Larson, 1998; Dorchin et al., 2006; Patankar et al., 2011). Cynipid wasp galls formed by *Antistrophus silphii* on *Silphium integrifolium* increased photosynthesis, stomatal conductance, and xylem water potential (Fay et al., 1993). In *Acacia pycnantha*, photosynthetic rates in phyllodes subtending clusters of galls by *Trichilogaster signiventris* were greater than rates in control phyllodes. However, a gall-inducing arthropod *Vasates aceriscrumena* caused declines in canopy photosynthetic rate and stomatal conductance in plants of *Acer saccharum* (Patankar et al., 2011). We found that the infection of *L. invasa* led to declines in leaf chlorophyll content, CO_2_ assimilation rate and photosynthetic electron flow. For a given plants grown at the same environmental conditions, leaf chlorophyll content is positively correlated with leaf nitrogen content (Yamori et al., 2011). For example, tobacco plant grown with high nitrogen concentration had higher leaf N content and chlorophyll content (Yamori et al., 2011). Therefore, the lower chlorophyll content in leaves of galled plants of *E. camaldulensis* indicated that gall wasp significantly decreased leaf N content. Nitrogen supply to leaf is dependent on stem and leaf hydraulic conductance. In galled plants, *K_stem_* and *K*_leaf_ were restricted by the larvae of *L. invasa*. Furthermore, the development of larvae in the cortex of shoots or the midribs of leaves needs lots of organic matters including N, which further decreases the N supply in leaves. The decline in leaf nitrogen content induces a decrease in RuBisCO content and thus declines in CO_2_ assimilation rate and photosynthetic electron flow. For the infected plants, the rate of CO_2_ assimilation under high light was significantly lower than the non-galled plants. What is more, the galled plants have much lower leaf number, which largely decreases the primary productivity of whole plants, thereby slowed the growth of plants.

In summary, we found that the stem and leaf hydraulic conductance significantly decreased in the galled plants due to development of larvae in the cortex of shoots or the midribs of leaves. Subsequently, the development of stomata and MVD were affected. To balance water supply and transpiration, leaves of galled plants had lower SD and g_s_. In addition, transpiration in the galled plants was further controlled due to decreased leaf number and total leaf area. The decreased photosynthetic rate and lower leaf number restricted the primary productivity in galled plants. The present study strongly indicated that *L. invasa* largely declined the hydraulic conductance and then depressed the growth of *E. camaldulensis*.

## Author Contributions

Conceived and designed the experiments: WH, Y-GT, and X-XD. Performed the experiments: WH, K-CZ, and XY. Analyzed the data: WH and YGT. Wrote the paper: WH, Y-GT, and X-XD.

## Conflict of Interest Statement

The authors declare that the research was conducted in the absence of any commercial or financial relationships that could be construed as a potential conflict of interest. The reviewer ZN and handling Editor declared their shared affiliation, and the handling Editor states that the process nevertheless met the standards of a fair and objective review.

## Supplementary Material

FIGURE S1 **Representative paradermal sections from leaves of galled and non-galled plants.** **(A)** abaxial side of non-galled plants; **(B)** adaxial side of non-galled plants; **(C)**, abaxial of galled plants; **(D)** adaxial side of galled plants.
