# Supplementary figures and images for: Effect of the Gall Wasp Leptocybe invasa on Hydraulic Architecture in Eucalyptus camaldulensis Plants
Source: Front Plant Sci. 2016 Feb 15;7:130. doi: 10.3389/fpls.2016.00130 (PMC4753697; doi:10.3389/fpls.2016.00130)

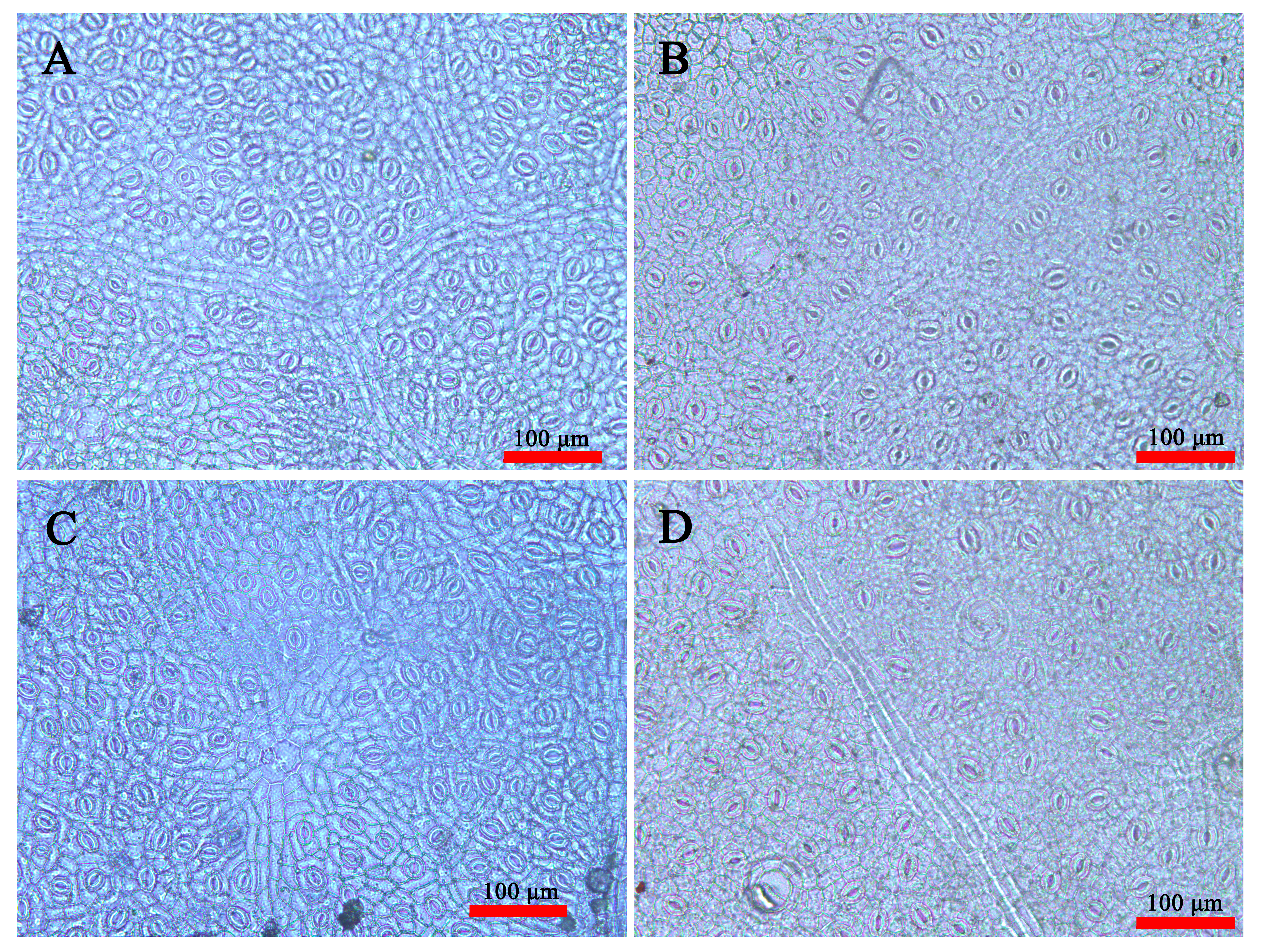

Supplement: FIGURE S1 — Representative paradermal sections from leaves of galled and non-galled plants. (A) abaxial side of non-galled plants; (B) adaxial side of non-galled plants; (C), abaxial of galled plants; (D) adaxial side of galled plants. [file Image_1.JPEG]
